# Efficacy of Adjunctive Photobiomodulation in Patellofemoral Pain Syndrome: A Systematic Review and Meta-Analysis

**DOI:** 10.1016/j.arrct.2026.100585

**Published:** 2026-01-16

**Authors:** Hongxiu Chen, Somkiat Asawaphureekorn, Nuttaset Manimmanakorn, Xingze Wang, Wichai Eungpinichpong

**Affiliations:** a Department of Exercise and Sport Sciences, Graduate School, Khon Kaen University, Khon Kaen, Thailand; b Department of Ophthalmology, Faculty of Medicine, Khon Kaen University, Khon Kaen, Thailand; c Department of Rehabilitation Medicine, Faculty of Medicine, Khon Kaen University, Khon Kaen, Thailand; d School of Physical Education, Huzhou University, Zhejiang Province, China; e Research Center in Back, Neck, Other Joint Pain and Human Performance (BNOJPH), Division of Physical Therapy, Faculty of Associated Medical Sciences, Khon Kaen University, Khon Kaen, Thailand; f School of Physical Therapy, Faculty of Associated Medical Sciences, Khon Kaen University, Khon Kaen, Thailand

**Keywords:** Low-level laser therapy, Meta-analysis, Patellofemoral pain syndrome, Photobiomodulation, Rehabilitation

## Abstract

**Objective:**

To evaluate the effectiveness and safety of adjunctive photobiomodulation (PBM) or low-level laser therapy (LLLT) combined with exercise-based rehabilitation in reducing pain and improving function in individuals with patellofemoral pain syndrome (PFPS).

**Data Sources:**

PubMed, Scopus, Embase, Web of Science, PEDro, the Cochrane Library, and China National Knowledge Infrastructure were searched from inception to October 2025.

**Study Selection:**

Randomized controlled trials (RCTs) were included if they examined PBM/LLLT with clearly reported parameters compared with placebo, sham, or no-intervention controls in individuals with PFPS. Studies with mixed knee pathologies or nonrandomized designs were excluded.

**Data Extraction:**

Two reviewers independently extracted data on participant features, PBM parameters, interventions, comparators, outcomes, and adverse events. Risk of bias was assessed using the Cochrane tool, with disagreements resolved by consensus.

**Data Synthesis:**

Four RCTs (n=162) were eligible. Meta-analysis using a random-effects model showed a statistically nonsignificant trend favoring PBM for pain reduction (effect size=−1.520; 95% confidence interval: −3.542 to 0.502; *P*=.097), with substantial heterogeneity (I^2^=91.1%) and a wide 95% prediction interval (−5.880 to 2.840). Importantly, the 4 included trials used distinct PBM delivery approaches, including laser acupuncture, PBM combined with trigger point therapy, classic PBM, and monochromatic infrared energy. This diversity may limit the validity of pooling these heterogeneous interventions within a single meta-analysis. Individually, PBM produced greater short-term pain reduction and some functional improvements in certain trials. No adverse events were reported.

**Conclusions:**

PBM/LLLT may provide short-term pain relief and modest functional benefits for young adults with PFPS and appears to be safe. However, the considerable variability among PBM modalities, including acupuncture-based laser stimulation, PBM combined with trigger point therapy, conventional PBM, and monochromatic infrared energy, introduces important methodological heterogeneity and raises concerns regarding the appropriateness of aggregating these interventions. In addition, the limited sample sizes and heterogeneous PBM protocols reduce confidence in these findings. Larger, standardized RCTs are needed.

Patellofemoral pain syndrome (PFPS) is a chronic musculoskeletal condition characterized by pain in the anterior knee or around the patella.^1^ It is commonly observed in adolescents, athletes, and individuals who frequently engage in activities involving the knee, and is one of the leading causes of anterior knee pain (AKP).^2^ The prevalence in the general population is approximately 22.7%, rising to 28.9% among adolescents, with females affected roughly twice as often as males.^3^ The pain is typically exacerbated during activities such as climbing stairs, squatting, prolonged knee flexion, or running, and is relieved with rest.^4^ Chronic pain and functional limitations associated with PFPS can significantly impair physical performance and quality of life.^5^

The pathogenesis of PFPS is complex and is primarily associated with abnormal patellar tracking within the femoral trochlea, lower limb malalignment, imbalances of the periarticular musculature, tension in joint soft tissues, excessive physical activity, and microdamage to the cartilage.^2^^,^^6^ The therapeutic goals are to relieve pain, improve knee joint function, and restore normal movement patterns. Conservative management of PFPS mainly involves muscle strengthening and stretching exercises.

Multiple studies have demonstrated that combined training of the quadriceps and hip abductors is more effective in alleviating pain, enhancing knee function, and reducing patellofemoral joint stress than isolated quadriceps exercises.^7^^,^^8^ Additional interventions include knee braces or patellar straps, physical modalities (eg, neuromuscular electrical stimulation, biofeedback, transcutaneous electrical nerve stimulation, heat therapy, and ultrasound), oral anti-inflammatory or analgesic medications, activity modification, and appropriate footwear.^9^^,^^10^

Despite exercise therapy being considered the criterion-standard conservative treatment for PFPS,^11^ its clinical effectiveness is often constrained by pain. Many patients report pain levels that hinder full participation in strengthening and functional training, leading to suboptimal adherence and delayed recovery. In the early phase of rehabilitation, when pain is most pronounced, clinicians frequently seek noninvasive adjuncts that can provide rapid analgesia without significant adverse effects, thereby serving as a “bridge” to enable patients to tolerate and comply with active exercise programs. This clinical gap highlights the need for adjunctive modalities that can safely reduce pain, facilitate load tolerance, and enhance the overall effectiveness of rehabilitation. Photobiomodulation (PBM), previously known as low-level laser therapy (LLLT), has been applied in pain and musculoskeletal rehabilitation since the late 1960s, with early clinical evidence supporting its analgesic effects.^12^

PBM is defined as the therapeutic use of nonionizing light (typically in the red or near-infrared spectrum) delivered by low-power laser or light-emitting diode (LED) devices to modulate biological processes. Thus, PBM encompasses both laser and LED-based interventions, whereas the historical term LLLT refers more narrowly to laser sources.^13^ PBM delivers low-intensity light irradiation of varying wavelengths to the affected area, harnessing photobiomodulatory effects to promote tissue repair, reduce inflammatory responses, and alleviate pain. Several studies have demonstrated its potential efficacy in chronic conditions such as osteoarthritis and tendinitis.^14^, ^15^, ^16^

The underlying mechanisms are thought to involve absorption of red or near-infrared light by mitochondria, leading to enhanced adenosine triphosphate (ATP) synthesis and cellular signaling, promotion of cell proliferation and stem cell differentiation, upregulation of growth factor production, attenuation of oxidative stress, and activation of ATP resynthesis through membrane hyperpolarization, thereby facilitating tissue repair and regeneration.^17^, ^18^, ^19^ In particular, cytochrome c oxidase, which represents complex IV of the mitochondrial respiratory chain, has been proposed as a primary photoacceptor for PBM, mediating increases in mitochondrial membrane potential, ATP production, and downstream signaling cascades that modulate inflammation and nociception.^13^^,^^20^, ^21^, ^22^ These biophysiological effects provide a plausible rationale for using PBM as an adjunctive modality to reduce pain and improve function in PFPS by targeting peripatellar soft tissues and neuromuscular control.

However, although PBM has shown promise in other musculoskeletal conditions, its specific role as an adjunct to exercise-based rehabilitation in PFPS remains unclear. Existing randomized controlled trials (RCTs) have employed heterogeneous PBM protocols (eg, different wavelengths, energy densities, treatment sites, and delivery modes such as laser acupuncture or combined laser/LED clusters), making it difficult to draw firm conclusions or establish standardized clinical guidelines.

This review aims to systematically summarize the evidence on the application of PBM/LLLT combined with rehabilitation training in PFPS, analyze its potential mechanisms of action, and highlight gaps in current research regarding population coverage, intervention standardization, efficacy follow-up, and mechanistic validation, thereby providing guidance for future studies and clinical practice.

## Methods

This systematic review was pre-registered in PROSPERO (CRD420251237649), with the protocol detailing the study objectives, methodology, and planned analyses to ensure transparency and consistency. The conducting and reporting of this systematic review followed the PRISMA-P and PRISMA guidelines.

### Data extraction

**Fig 1:**
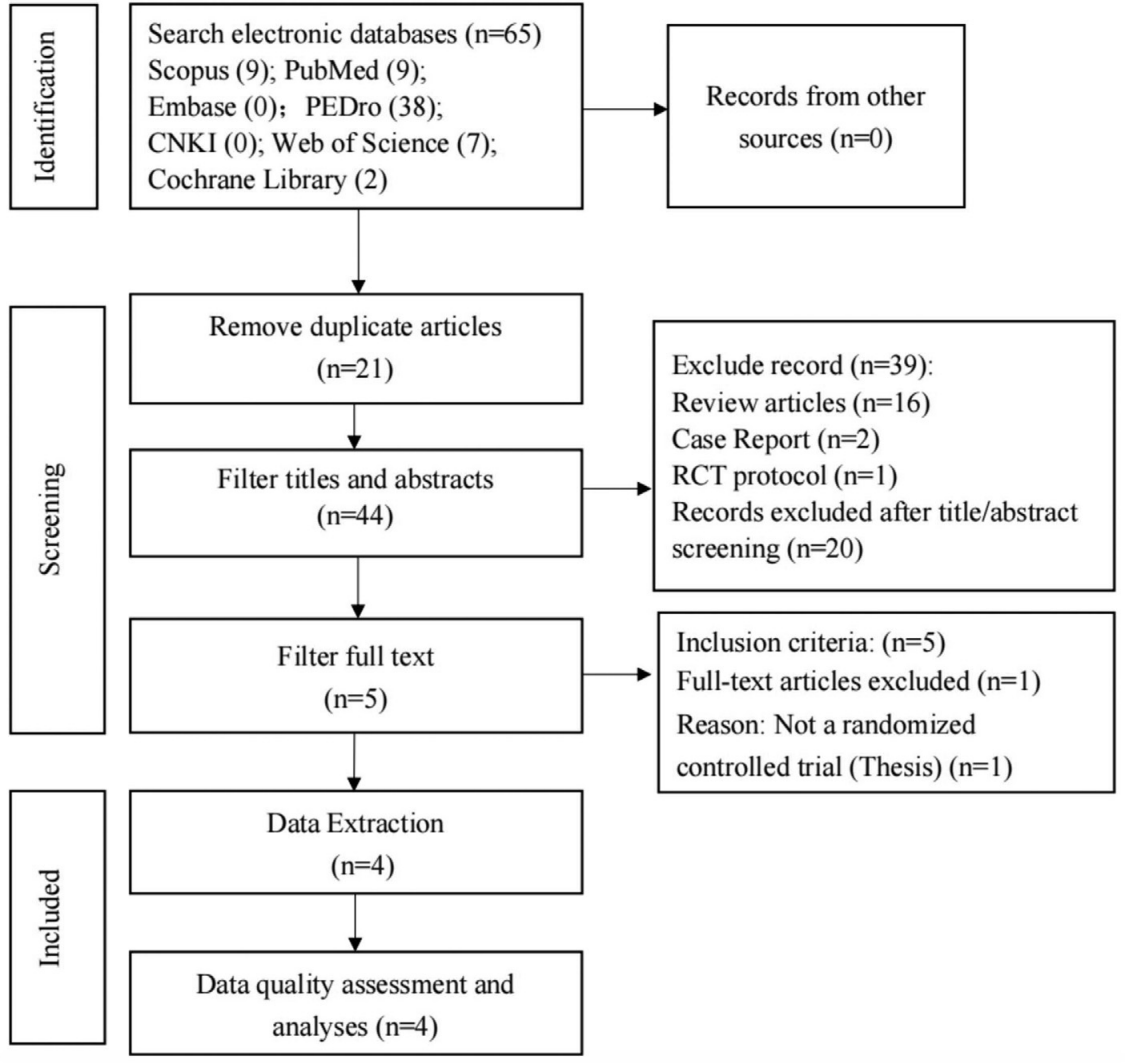
Literature screening flowchart.

This systematic review followed established guidelines for conducting and reporting systematic reviews. A comprehensive search was performed across multiple electronic databases, including PubMed, Scopus, Embase, PEDro, Web of Science, the Cochrane Library, and China National Knowledge Infrastructure (CNKI), covering all available records. As illustrated in the Preferred Reporting Items for Systematic Reviews and Meta-Analyses (PRISMA) flowchart (fig 1), a total of 65 records were identified through database searching, with no additional records retrieved from other sources. After removing 21 duplicates, 44 records remained for title and abstract screening. During this stage, 39 records were excluded, including 16 review articles, 2 case reports, 1 RCT protocol, and 20 articles excluded based on title/abstract screening. Subsequently, 5 full-text articles were assessed for eligibility. Of these, 1 full-text record was excluded because it was a thesis rather than a peer-reviewed academic article and did not meet the requirement of being an RCT. Ultimately, 4 studies met all inclusion criteria and were included for data extraction, methodological quality assessment, and final analysis.

A comprehensive search strategy combining Medical Subject Headings terms and free-text keywords was employed. The search included condition-related terms such as “patellofemoral pain syndrome,” “patellofemoral pain,” “anterior knee pain,” “retropatellar pain,” and “chondromalacia patellae,” as well as intervention-related terms including “low-level laser therapy,” “low level laser therapy,” “laser therapy,” and “photobiomodulation.” In addition, the reference lists of all included studies were manually screened to identify any relevant articles that may have been missed during the electronic database searches. All retrieved records were imported into EndNote 20 for reference management, and duplicate entries were removed before the screening process.

A comprehensive search was conducted in PubMed using the following keywords: (“patellofemoral pain syndrome” OR “patellofemoral pain” OR “anterior knee pain” OR “retropatellar pain” OR “chondromalacia patellae”) AND (“low-level laser therapy” OR “low level laser therapy” OR “photobiomodulation” OR “laser therapy”). The search included all languages and covered publications from database inception to October 2025. Retrieved records were exported to EndNote for duplicate removal, and titles and abstracts were screened for relevance, followed by full-text review to determine eligibility. The entire process was independently conducted by 2 reviewers, with disagreements resolved through discussion or consultation with a third reviewer when necessary.

To maximize internal validity, we applied strict eligibility criteria and excluded nonrandomized designs, studies with mixed or poorly defined knee pathologies, and trials in which PBM was combined with other nonstandard cointerventions that could confound the effects of PBM itself. This “quality over quantity” approach likely contributed to the small number of included studies but ensured that only high-quality RCTs were pooled in the meta-analysis.

### Deviations from the protocol

To ensure the reliability of the results, the Cochrane Risk of Bias Tool was still applied to assess the risk of bias in included RCTs,^23^ systematically identifying potential sources of bias that could affect the conclusions. No major deviations from the preregistered protocol were made in terms of study design, eligibility criteria, or primary outcomes. The main unplanned adaptation was a more narrative synthesis of secondary outcomes and mechanistic findings, because of the limited number of eligible RCTs and the heterogeneity of functional outcome measures.

Two independent reviewers (H.C. and N.M.) screened the titles and abstracts of all identified articles according to predefined inclusion criteria to determine eligibility for full-text review. Any disagreements during the screening process were resolved through discussion and, when necessary, by consultation with a third reviewer (W.E.).

### Inclusion and exclusion criteria

This review included all studies that investigated LLLT combined with rehabilitation training in patients with PFPS, regardless of study design. Participants were required to meet the following criteria: they must have been diagnosed with PFPS or presented with typical symptoms of anterior or retropatellar knee pain. Studies were excluded if participants had other knee disorders, such as ligament or meniscal injuries, patellar tendinopathy, recurrent patellar subluxation, radiographically confirmed osteoarthritis, or were awaiting surgery for PFPS. Studies published in all languages were included. For the quantitative synthesis, only RCTs with a clearly described PBM protocol (including wavelength, power or irradiance, and treatment schedule) and a control or sham comparator were included.

### Quality assessment

Two independent reviewers (H.C. and S.A.) assessed the methodological quality of the included studies using the Cochrane Risk of Bias Tool,^23^ which is designed for evaluating interventional studies and explicitly appraises each domain of bias.^24^ The tool evaluates 7 domains: selection bias (random sequence generation and allocation concealment), performance bias (blinding of participants and personnel), detection bias (blinding of outcome assessors), attrition bias (incomplete outcome data), reporting bias (selective outcome reporting), and other sources of bias (eg, funding, baseline imbalances). Each domain was rated as low, high, or unclear risk of bias. The overall risk of bias for each study was determined as follows: studies with all domains rated low were considered low overall risk; studies with all domains rated low or unclear and no domain rated high were judged as unclear overall risk; and studies with any domain rated high were classified as high overall risk.^23^ Discrepancies were resolved through consensus meetings, with unresolved disagreements adjudicated by a third reviewer (X.W.). Detailed results are presented in table 1.

**Table 1:** Cochrane risk of bias assessment

References	Random Sequence Generation	Allocation Concealment	Blinding of Participants and Personnel	Blinding of Outcome Assessment	Incomplete Outcome Data	Selective Reporting	Other Bias	Overall Offset Risk
Gavish et al.^25^	Low risk	Low risk	Low risk	Low risk	Low risk	Low risk	Low risk	Low risk
Allam et al.^26^	Low risk	Low risk	Low risk	Low risk	Low risk	Low risk	Low risk	Low risk
Pocai et al.^27^	Low risk	Low risk	Low risk	Low risk	Low risk	Low risk	Low risk	Low risk
Alrawaili et al.^28^	Low risk	Low risk	High risk	Low risk	Low risk	Low risk	Low risk	High risk

### Data management and analysis

The included studies were independently reviewed by 2 researchers (H.C. and S.A.) using a standardized data extraction form. Extracted information included study characteristics, participant profiles, intervention parameters (PBM/LLLT modalities and rehabilitation protocols), comparators, follow-up durations, outcomes (eg, pain, knee function, range of motion), and adverse events. All data were cross-checked by a third researcher (X.W.) to ensure accuracy. Study characteristics are summarized in table 2.

**Table 2:** Characteristics of included studies

Author (Year)	Design	Sample	Intervention	Control	Main Outcome
Gavish et al.^25^	RCT	26 soldiers/police (M:11,F:15) with AKP (age >18 y)	PBM was delivered using a combination of light-emitting diode (LED) therapy (660/850 nm, 50 mW/cm^2^) for anti-inflammatory effects and laser therapy (810 nm, 4.75-6 W/cm^2^) targeting deep muscle trigger points and L2-L5 nerve roots for analgesia. Treatments were applied as single-point and 5 × 810 nm cluster approaches, combined with physiotherapy, for 8 sessions over 4 wk	Sham PBM+physiotherapy	The PT+PBM group demonstrated greater improvements than the control group in knee pain relief, functional outcomes, and long-term satisfaction. The intervention was safe, and no adverse events were reported
Allam et al.^26^	RCT	60 young women (age 18-25 y)	Laser acupuncture (LA) at 6 knee acupoints (905 nm, 100 mW, 4 J/point, 80 s/point)+exercise program, 2 × /wk for 4 wk	Sham LA (device off)+same exercise program, 2 × /wk for 4 wk	The real laser acupuncture group demonstrated significantly greater improvements than the sham laser group in pain reduction and knee joint range of motion (ROM), as measured by the Visual Analog Scale (VAS), ROM, and Kujala scores
Pocai et al.^27^	RCT	30 women with PFPS (age 21.87±2.74 y)	Photobiomodulation (PBM: cluster of infrared laser [830 nm]+amber LED [590 nm], 12 sessions over 4 wk, 3 × /wk, lateral and medial patella)	No intervention, only assessments	PBM reduced landing pain (VAS) but did not improve functional tests; Knee Injury and Osteoarthritis Outcome Score (KOOS) and Anterior Knee Pain Scale (AKPS) showed larger (nonsignificant) effect sizes favoring PBM
Alrawaili et al.^28^	RCT	46 adolescents with PFPS (age: 15-18 y)	MIRE+PT (12 wk): standard physical therapy (3 × /wk, 60 min/session; hip and knee stretching and strengthening, balance training, ultrasound and heat) plus monochromatic infrared energy delivered via Anodyne Therapy Professional Infrared Therapy System (Model 480; 8 diode-array pads placed medially, laterally, anteriorly, and posteriorly on both knees; 890 nm wavelength; total radiant power 6.24 W; 40 min/session, 3 × /wk for 12 wk)	Standard PT program only (same frequency/duration as above), without MIRE	MIRE+PT vs PT: lower post-treatment VAS (2 [2-3] vs 4 [4-5], *P*<.001), better mSEBT (all *P*≤.019), higher Kujala (*P*=.008), and higher PedsQL physical, psychosocial, and total scores (*P*≤.045)

Before conducting the quantitative synthesis, we evaluated whether pooling the data were conceptually and clinically appropriate. Although the included studies applied different PBM delivery methods, such as conventional PBM, laser acupuncture, and monochromatic infrared energy (MIRE), these techniques operate through the same fundamental therapeutic mechanism by using near-infrared or infrared light to modulate cellular activity, reduce inflammation, and relieve pain. Therefore, they were regarded as different forms of a single core intervention.

**Fig 2:**
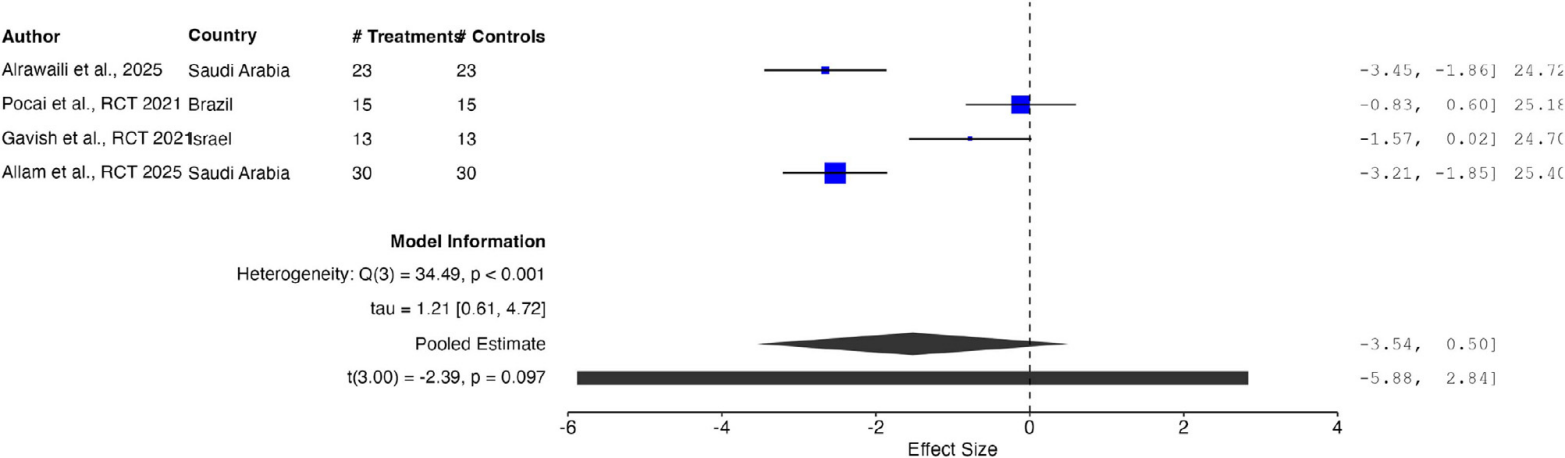
Forest plot of the 4 included randomized controlled trials comparing PBM/LLLT versus placebo. Squares indicate the effect size of individual studies, whereas the diamond represents the pooled effect. Considerable heterogeneity was observed (I^2^=91.1%), which may be attributed to the variations in effect sizes between the studies, particularly the larger effects reported in the recent 2025 trials.

A random-effects model was used to calculate pooled effect estimates for outcomes reported by sufficiently homogeneous subsets of studies. Statistical heterogeneity was quantified using the I^2^ statistic and τ^2^, and a 95% prediction interval was calculated to describe the expected range of true effects in future clinical settings. Results of the meta-analysis are presented in figure 2, and additional findings and mechanisms were synthesized narratively.

## Results

### Meta-analysis results

Four RCTs were included in the quantitative synthesis. The pooled analysis demonstrated an overall effect size of −1.520, indicating a trend favoring PBM. However, this effect was not statistically significant (95% confidence interval: −3.542 to 0.502; *P*=.097). Substantial heterogeneity was observed across studies (I^2^=91.1%, τ^2^=1.473), indicating considerable variability in treatment effects. The wide 95% prediction interval (−5.880 to 2.840) further suggested that the true effects in future similar studies may vary substantially. Given the high heterogeneity, these pooled results should be interpreted with caution. Characteristics of the included studies are presented in table 2, and the pooled effects are shown in figure 2.

### Summary of individual study findings

This systematic review synthesized evidence from 4 RCTs evaluating the effects of LLLT or PBM on PFPS or AKP. As summarized in table 2, all included studies were published between 2021 and 2025, with a total of 162 participants aged 15-25 years. Sample sizes ranged from 26 to 60, and women represented the majority in 3 of the studies. The interventions varied substantially and included PBM delivered through combined LED and laser light (660/850 nm and 810 nm), laser acupuncture using a 905-nm laser, a cluster of infrared laser (830 nm) combined with amber LED (590 nm), and MIRE. Treatment frequency ranged from 4 to 36 sessions over 4-12 weeks, and PBM was commonly combined with physiotherapy or structured exercise programs. Control conditions included sham laser procedures, physiotherapy alone, or no active intervention.

Across the 4 included trials, PBM, laser acupuncture, or MIRE showed a trend toward greater pain reduction compared with control conditions; however, this effect was not statistically significant (Visual Analog Scale, pooled *P*=.097). Although the overall pooled effect did not reach statistical significance because of high heterogeneity and the limited number of studies, it is noteworthy that 2 individual trials 26, 28 reported statistically significant pain relief in favor of the PBM group.

## Discussion

As far as we know, this is the first systematic review and meta-analysis specifically evaluating the therapeutic effects of PBM/LLLT on PFPS in young adults. The primary findings from our quantitative synthesis of 4 RCTs (n=162) demonstrated a large effect size favoring PBM for pain reduction (Standardized Mean Difference=−1.520); however, this result did not reach statistical significance (*P*=.097; 95% confidence interval:−3.542 to 0.502).

The substantial heterogeneity observed (I^2^=91.1%) is a critical factor in interpreting these results. This variability stems primarily from the lack of standardization in PBM parameters, including differences in wavelength (590-905 nm), energy density, and treatment frequency (4-36 sessions). In addition, a notable methodological limitation was the pooling of clinically distinct intervention types, including classic PBM, laser acupuncture, and MIRE. This methodological diversity likely exacerbated the observed heterogeneity and reduced the comparability of the findings across studies.

Mechanistically, PBM exerts therapeutic effects by enhancing mitochondrial cytochrome c oxidase activity and ATP production.^13^, ^15^, ^29^, ^30^, ^31^, ^32^ It may also modulate inflammatory mediators and reduce nociceptive neuron excitability, thereby decreasing peripatellar sensitivity.^33^, ^34^, ^35^, ^36^ In clinical practice, these physiological benefits could alleviate exercise-induced discomfort, potentially improving patient adherence to standard strengthening protocols.^37^^,^^38^ However, although our findings align with a general positive trend in adjunctive PBM research,^15^^,^^39^ the current lack of statistical significance, which is likely attributable to the small total sample size and protocol inconsistencies, precludes a definitive clinical recommendation.

### Limitations and future directions

This review has several limitations. First, the small number of eligible RCTs (n=4) and the modest sample sizes (26-60 participants) reduced statistical power and prevented the conduct of subgroup or sensitivity analyses. Second, a key methodological limitation was the pooling of clinically diverse PBM-based interventions, including classic PBM, laser acupuncture, and MIRE. This heterogeneity in treatment modality, together with marked inconsistencies in parameters such as wavelength, energy dose, and treatment frequency, likely contributed to the substantial statistical heterogeneity observed (I^2^=91.1%). Third, the lack of long-term follow-up data and the focus on adolescents and young adults limit the ability to determine the durability of treatment effects and restrict the generalizability of the findings to older or more clinically diverse populations.

Future research should prioritize large-scale, multicenter RCTs using standardized PBM protocols and core outcome sets for PFPS. Detailed reporting of dose parameters (wavelength, irradiance, and fluence) and mechanistic substudies (eg, inflammatory biomarkers) are essential to link PBM’s molecular effects to clinical outcomes. Despite these limitations, current evidence suggests that PBM/LLLT remains a safe, potential adjunctive therapy for short-term pain relief.

## Conclusion

This systematic review and meta-analysis synthesized evidence from 4 RCTs evaluating the effects of LLLT or PBM on PFPS or AKP. Critically, the overall pooled effect for pain reduction did not reach statistical significance (*P*=.097), and substantial heterogeneity was observed across the included studies (I^2^=91.1%). Although individual trials and effect size trends suggest that PBM/LLLT may provide short-term pain relief and functional benefits, the lack of statistical power in the pooled analysis precludes a definitive endorsement of its efficacy. No serious adverse events were reported, suggesting the intervention is generally safe. In conclusion, although PBM/LLLT shows potential as a safe adjunctive therapy, it cannot yet be recommended as a routine treatment for PFPS/AKP because of the limited number of studies, small sample sizes, and significant variability in protocols. Future research should prioritize large-scale, well-designed multicenter RCTs with standardized protocols to confirm these preliminary trends.

## Disclosures

None.
